# Extracellular volume(ECV) quantified by T1 mapping could reflect effect of long term blood pressure control status in patients with essential hypertension

**DOI:** 10.1186/1532-429X-18-S1-P125

**Published:** 2016-01-27

**Authors:** Xuefei Duan, Yucheng Chen, Tianjing Zhang

**Affiliations:** 1 Sichuan Province People's Hospital, Chengdu, China; 2 grid.412901.f0000000417701022Cardiology Department, Sichuan University West China Hospital, Chengdu, China; 3 MR Collaborations NE Asia, Siemens Healthcare, Beijing, China

## Background

Diffuse myocardial fibrosis is an important pathophysiological process involved into essential hypertension which may increase myocardium stiffness, cause ventricular remodeling and increase adverse cardiovascular risk eventually. It is strongly evident that optimal antihypertensive therapy will reduce the risk induced by hypertension, however, very few proportion of patients with hypertension can reach the recommended target of blood pressure (BP) control. So far little knowledge has been illuminated for effect of blood pressure control status on the myocardial fibrosis in hypertension. T1 mapping and extracellular volume (ECV) quantified by CMR has been adopted as a good method in examination of diffuse fibrosis. Besides, variation of serum fibrosis biomarkers may also reflect the degree of myocardial fibrosis. Based on the above we hypothesised that there was a correlation between ECV, serum fibrotic biomarkers and long-term BP control status in patients with essential hypertension.

## Methods

T1 mapping by using modified Lock-Locker inversion recovery(MOLLI) sequence on a 3.0 Tesla scanner was performed on 40 hypertension patients (20 patients with documented well BP control and 20 with uncontrolled BP) and 20 healthy controls. Myocardial native, post T1 and average ECV of left ventricle were measured based on pre contrast and post contrast T1 mapping(15 minutes after intravenous injection with gadopentetate dimeglumine (0.15 mmol/kg). Serum PICP and ICTP level were detected with ELISA kits.

## Results

Uncontrolled hypertensive patients had a higher interventricular septum thickness(IVS) or left ventricular mass index(LVMI) than well controlled hypertensive patients, whereas there was no difference in left ventricular functional parameters between two groups. Hypertensive patients had higher mean ECV of left ventricular myocardium than normal controls, furthermore, mean ECV of LV in hypertensive patients with uncontrolled BP was higher than in patients with ECV well- controlled(29.4 ± 3.1 vs. 27.6 ± 2.1, p = 0.01),whereas the differences of native or post-contrast T1 time of LV myocardium between the two groups was not statistically significant (p = 0.75 and 0.19, respectively)(Table 1). By multi variants analysis, LVMI was independently correlated with ECV. PICP and the ratio of PICP/ICTP showed a trend towards higher in hypertension patients with uncontrolled BP than well-controlled (76.6 ± 13 ug/L vs. 66.82 ± 15.3 ug/L, p = 0.59 and 73.22 ± 13.6 vs. 66.82 ± 15.3, p = 0.96, respectively), and it was proved that there were correlations between increased ECV and elevated serum fibrosis biomarkers level (PICP: *β* = 0.37, p = 0.02, PICP/ICTP: *β* = 0.30, p = 0.03) (Table 2).

**Table 1:** Cardiac Magnetic Resonance Imaging data.

	Well-controlled BP(n = 20)	Uncontrolled BP (n = 20)	Control (n = 20)	P value
LVEDV, ml	112.7 ± 23.8	121.6 ± 19.7	109.3 ± 17.8	>0.05
LVESV, ml	40.7 ± 9.2	46.3 ± 1.4	39.8 ± 1.0	>0.05
LVEDVI, ml/BSA	67.5 ± 11.9	68.1 ± 12.1	68.3 ± 10.7	>0.05
LVESVI, ml/BSA	24.5 ± 4.9	25.6 ± 7.9	25.6 ± 6.4	>0.05
LVM, g	75.8 ± 15.6*	82.1 ± 17.4*	63.8 ± 12.8	<0.05
LVMI, g/BSA	45.4 ± 8.2	46.0 ± 9.6	41.8 ± 9.5	>0.05
IVS, mm	8.7 ± 1.0*	9.9 ± 2.7**#	7.4 ± 1.2	<0.001
LVEF, %	63.5 ± 5.7	61.6 ± 6.7	63.1 ± 7.1	>0.05
SV, ml	71.8 ± 18.3	74.4 ± 12.5	68.1 ± 13.1	>0.05
Native myocardium T1 time, msec	1257 ± 61	1271 ± 58	1233 ± 66	>0.05
Post-contrast T1 time, msec	461 ± 43	443 ± 47	474 ± 44	>0.05
ECV, %	27.6 ± 2.1*	29.4 ± 3.1**#	26.1 ± 1.6	<0.001

Values are mean ± SD

LVEDV = left ventricular end-diastolic volume; LVESV = left ventricular end-systolic volume; BSA = body surface area; LVEDVI = left ventricular end-diastolic volume index; LVESVI = left ventricular end-systolic volume index; LVM=left ventricular mass; LVMI=left ventricular mass index; IVS= interventricular septum; LVEF = left ventricular ejection fraction; SV= stroke volume; ECV = extracellular volume fraction;

* p <0.05 vs.control, ** p <0.001 vs.control, # p <0.05 vs. Well-controlled BP group (LSD post-hoc tests for differences)

**Table 2:** Relationship of serum fibrosis biomarkers with demographic T1 mapping data

	PICP				ICTP		PICP/ICTP			
	r	P value	β	P value	r	P value	r	P value	β	P value
Age, yrs	0.20	0.11			0.14	0.26	0.10	0.44		
BMI, kg/m2	0.01	0.94			0.14	0.29	0.23	0.07		
SBP, mmHg	0.15	0.25			0.18	0.16	0.25*	0.04	-0.19	0.32
DBP, mmHg	0.28*	0.03	0.11	0.46	0.17	0.18	0.27*	0.03	0.36	0.06
LVEF, %	0.20	0.12			0.16	0.23	-0.16	0.20		
LV mass, g	0.18	0.15			0.28*	0.03	-0.07	0.68		
IVS, mm	0.27*	0.03	-0.05	0.78	0.04	0.80	0.24	0.06		
Native T1 time, msec	0.16	0.21			0.01	0.92	0.16	0.20		
Post-contrast T1 time, msec	0.05	0.67			-0.08	0.53	0.10	0.42		
ECV, %	0.4*	0.01	0.37*	0.02	0.07	0.60	0.37*	0.003	0.30*	0.03

Values are mean ± SD.

PICP: carboxy-terminal propeptide of procollagen type I; ICTP: carboxy-terminal telopeptide of collagen type I; BMI = body mass index; SBP = systolic blood pressure, DBP = diastolic blood pressure; LVEF = left ventricular ejection fraction; IVS = interventricular septum; ECV = extracellular volume fraction.

* p <0.05.

## Conclusions

ECV of LV myocardium was higher in hypertensive patients which was associated with BP control status. ECV could be associated with the LV hypertrophy in patients with uncontrolled hypertension and could also reflect the changes of myocardial fibrosis in hypertension.

